# Impact of the COVID-19 pandemic on patients with rheumatoid arthritis: data from the Ontario Best Practices Research Initiative (OBRI)

**DOI:** 10.1093/rap/rkad042

**Published:** 2023-04-26

**Authors:** Matthew Wong-Pack, Elliot Hepworth, Mohammad Movahedi, Bindee Kuriya, Janet Pope, Edward Keystone, Carter Thorne, Vandana Ahluwalia, Angela Cesta, Carol Mously, Claire Bombardier, Arthur Lau, Sibel Zehra Aydin

**Affiliations:** Division of Rheumatology, University of Toronto, Toronto, ON, Canada; Division of Rheumatology, University of Ottawa, Ottawa, ON, Canada; Toronto General Research Institute, University Health Network, Toronto, ON, Canada; Division of Rheumatology, University of Toronto, Toronto, ON, Canada; Division of Rheumatology, University of Western Ontario, London, ON, Canada; Department of Medicine, University of Toronto, Toronto, ON, Canada; Centre of Arthritis Excellence, Newmarket, ON, Canada; Division of Rheumatology, William Osler Health System, Brampton, ON, Canada; Toronto General Research Institute, University Health Network, Toronto, ON, Canada; Toronto General Research Institute, University Health Network, Toronto, ON, Canada; Division of Rheumatology, University of Toronto, Toronto, ON, Canada; Division of Rheumatology, McMaster University, Hamilton, ON, Canada; Division of Rheumatology, University of Ottawa, Ottawa, ON, Canada; The Ottawa Hospital Research Institute, Ottawa, ON, Canada

**Keywords:** COVID-19, coronavirus, patient-reported outcome, rheumatic diseases, RA, disease activity, medication adherence

## Abstract

**Objective:**

The coronavirus disease 2019 (COVID-19) pandemic created challenges for patients with RA. We examined the potential impact of the pandemic on patient-reported outcomes (PROs), disease activity and medication profiles, comparing the periods pre-pandemic and during the pandemic.

**Methods:**

Patients enrolled in the Ontario Best Practices Research Initiative were included if they had at least one visit to a physician or study interviewer within 12 months before and after the start of pandemic-related closures in Ontario (15 March 2020). Baseline characteristics, disease activity, PROs [i.e. health assessment questionnaire disability index, RA disease activity index (RADAI), European quality of life five-dimension questionnaire], medication use and changes were included. Student’s paired two-sample *t*-tests and McNamar’s tests were performed for continuous and categorical variables between time periods.

**Results:**

The sample for analysis consisted of 1508 patients, with a mean (s.d.) age of 62.7 (12.5) years, and 79% were female. Despite decreases in the number of in-person visits during the pandemic, there was no significant negative impact on disease activity or PRO scores. The DASs in both periods remained low, with either no clinically significant differences or slight improvement. Scores for mental, social and physical health were either stable or improved. There were statistically significant decreases in conventional synthetic DMARD use (*P* < 0.0001) and increased Janus kinase inhibitor usage (*P* = 0.0002). Biologic DMARD use remained stable throughout the pandemic.

**Conclusion:**

In this cohort, disease activity and PROs of RA patients remained stable during the COVID-19 pandemic. The longer-term outcomes of the pandemic warrant investigation.

Key messages

- Disease activity was generally stable during the pandemic period, similar to the pre-pandemic period.
- Patient-reported outcomes of patients before and during the COVID-19 pandemic were similar.
- Conventional synthetic DMARD use decreased, whereas Janus kinase inhibitor usage increased during the pandemic.

## Introduction

Coronavirus disease 2019 (COVID-19) has impacted health-care systems across the globe. This disease has negatively affected mental health, social well-being and the economy and has disrupted longitudinal patient care [1–4]. Patients with autoimmune rheumatic diseases might be at a particularly elevated risk for poorer outcomes when infected by COVID-19 owing to being immune suppressed from their illness and treatment [5].

Although much attention has been placed on the direct impact of the infection concerning morbidity and mortality for patients, research is necessary to evaluate how this pandemic has affected other aspects of patient care. A few studies with small sample sizes examining the mental health status of patients with rheumatic diseases compared with controls during the pandemic suggest that these patients had higher levels of distress and panic, with others showing increased depression and anxiety on a group level [6, 7]. How the pandemic impacted the patient-reported outcomes (PROs) in RA patients individually by comparing the periods before and during the pandemic is not fully elucidated.

The Ontario Best Practices Research Initiative (OBRI) is an organization founded in 2005 on the collaborative efforts between patients, rheumatologists and researchers to help improve the treatment and outcomes of Ontarians living with RA, currently with >3900 patients being followed prospectively [Ontario Best Practices Research Initiative (obri.ca)]. In this database, patients are approached regularly by the research team to collect multiple domains of PROs. We used this dataset to evaluate the impact of the COVID-19 pandemic by comparing patients’ pre-pandemic disease activity, medication profiles and PROs with data collected during the pandemic.

## Methods

### Study design

The OBRI is a multicentre provincial registry in Canada that collects data prospectively on RA patients followed in routine care. Patients eligible for inclusion in the registry must have a diagnosis of RA confirmed by a rheumatologist, disease onset ≥16 years of age, be ≥18 years of age at registry enrolment and have at least one swollen joint. Treating rheumatologists collect data through patient assessment during routine care, and patients also provide data directly via telephone interviews occurring every 6 months. The OBRI registry was established in accordance with the Declaration of Helsinki. Ethics approval was obtained for institutional sites (University Health Network Research Ethics Board no. 07-0729-AE) and approval at each participating site. Written informed consent was provided by all patients before enrolment in the registry.

### Study population

We defined two study periods, each of 1 year duration: a pre-COVID-19 pandemic phase (12 months before 15 March 2020) and a COVID-19 pandemic phase (12 months after 15 March 2020). We applied a time window period for data collection of ±60 days for 1 year before and during the COVID-19 pandemic (1 year + 60 days). Patients enrolled in OBRI were included if they had at least one visit to a physician or interviewer (virtually or in person) in both study periods. Patient characteristics, disease activity measurements, PROs, medication usage and the number of visits were recorded.

### Statistical analyses

Baseline characteristics, defined as the demographic data collected 1 year before the COVID-19 pandemic was declared in Ontario, were reported as the mean (s.d.) and percentages where appropriate. Student’s [aired two-sample *t*-tests and McNemar’s tests were performed for continuous and categorical variables, respectively, between the two study periods, with statistical significance set at <0.05.

## Results

The sample for analysis included 1508 patients who fulfilled the eligibility criteria. Of these, 1249 patients had at least one visit to a physician and 709 patients had at least one interview visit during both periods (Supplementary Fig. S1, available at *Rheumatology Advances in Practice* online). The mean (s.d.) age of patients was 62.7 (12.5) years; 1196 (79.3%) were female. The mean disease duration was 12.1 (10) years. The majority were seropositive, with either positive RF (70.9%) or positive ACPA (61.5%). The most common co-morbidities were hypertension (15.5%), diabetes (3.8%), respiratory disease (4.6%) and depression (5.3%).

In the pre-pandemic period, baseline disease activity was low, with the following scores reported [mean (s.d.)]: Clinical Disease Activity Index (CDAI) 8.66 (8.54), Simplified Disease Activity Index for Rheumatoid Arthritis (SDAI) 9.40 (8.95), DAS-28 2.97 (1.27) and RA Disease Activity Index (RADAI) 2.47 (1.95). Inflammatory markers were also low, with ESR of 19.3 (17.1) mm/h and CRP of 6.63 (11.0) mg/l. Patient-reported outcome measures were either low or moderate at baseline, with the following scores reported [mean (s.d.)]: Health Assessment Questionnaire-Disability Index (HAQ-DI) 0.91 (0.75), HAQ-pain 0.93 (0.76), fatigue 3.81 (2.87), sleep 2.92 (2.95), depression/anxiety 1.16 (0.35) and EQ5D EuroQoL 0.79 (0.17).

Table 1 highlights the physician-reported disease activity measures before and during the COVID-19 pandemic. Supplementary Fig. S2, available at *Rheumatology Advances in Practice* online, outlines the changes graphically. The number of physician visits per patient (virtual or in person) increased by an average of 0.21 (1.51). The number of days between visits decreased by 11.3 (66.9) days. Although improvements were noted in swollen and tender joint counts, there was no statistically significant difference in the overall patient global, physician global and some DASs (CDAI, SDAI and DAS). The ESR was elevated significantly when comparing laboratory investigations before and during the pandemic. There were statistically significant decreases in conventional synthetic DMARD (csDMARD) and CS use and increases in Janus kinase (JAK) inhibitor use between the two study periods. Use of biologic DMARDs (bDMARDs) remained unchanged between the two study periods.

**Table 1.** Physician disease activity measures 1 year before and during first year of coronavirus disease 2019 (15 March 2020)

Patients (*n* = 1249)	Within 1 year before COVID-19	Within first year of COVID-19	Paired comparison		
			Before	After	Difference, *P*-value
Number of visits per patient					
*n*	1249	1249	1249	1249	1249
Mean (s.d.)	2.21 (1.02)	2.42 (1.45)	2.21 (1.02)	2.42 (1.45)	0.21 (1.51), <0.0001
Total visits per patient					
*n*	1249	1249	–	–	–
One visit, *n* (%)	299 (23.9)	560 (44.8)	–	–	–
Two visits, *n* (%)	569 (45.6)	–	–	–	–
Three or more visits, *n* (%)	381 (30.5)	689 (55.2)	–	–	–
Time (days) between visits for patients with more than one visit including virtual visits					
*n*	947	686	578	578	578
Mean (s.d.)	148.2 (54.3)	131.1 (53.5)	139.7 (52.8)	128.4 (52.0)	−11.3 (66.9), <0.0001
Swollen joint count (0–10)					
*n*	1241	810	804	804	804
Mean (s.d.)	1.66 (2.67)	1.19 (2.67)	1.66 (2.80)	1.17 (2.67)	−0.49 (3.04), <0.0001
Tender joint count (0–10)					
*n*	1237	763	755	755	755
Mean (s.d.)	2.07 (3.30)	1.57 (3.22)	1.97 (3.20)	1.57 (3.22)	−0.40 (3.73), 0.003
Patient global assessment (0–10)					
*n*	1055	591	570	570	570
Mean (s.d.)	2.98 (2.41)	2.90 (2.41)	2.90 (2.42)	2.87 (2.41)	−0.03 (2.22), 0.77
Physician global assessment (0–10)					
*n*	1043	517	497	497	497
Mean (s.d.)	1.73 (1.82)	1.60 (1.85)	1.58 (1.80)	1.57 (1.83)	−0.01 (1.71), 0.90
Clinical disease activity index (0–76)					
*n*	1109	480	460	460	460
Mean (s.d.)	8.66 (8.54)	7.63 (8.68)	8.07 (8.15)	7.42 (8.54)	−0.65 (8.25), 0.09
Simplified disease activity index (0.1–86)					
*n*	949	369	342	342	342
Mean (s.d.)	9.40 (8.95)	8.43 (9.02)	8.47 (8.53)	8.31 (9.02)	−0.16 (8.68), 0.73
DAS-28 (0-9.4)					
*n*	991	440	401	401	401
Mean (s.d.)	2.97 (1.27)	2.91 (1.23)	2.82 (1.25)	2.89 (1.23)	0.07 (1.12), 0.19
ESR					
*n*	916	769	703	703	703
Mean (s.d.)	19.3 (17.1)	21.8 (19.1)	19.5 (17.5)	21.7 (19.1)	2.20 (1.29), <0.0001
CRP					
*n*	1051	917	835	835	835
Mean (s.d.)	6.63 (11.0)	6.55 (13.1)	6.63 (10.8)	6.34 (12.3)	−0.29 (11.5), 0.47
Medication number reported by physician					
*n*	1227	1216	1210	1210	1210
Mean (s.d.)	1.49 (0.64)	1.50 (0.65)	1.51 (0.63)	1.50 (0.65)	−0.01 (0.42), 0.37
bDMARDs use reported by physician					
Visits (*n*)	3101	3101	3101	3101	3101
Yes (%)	1119 (36.1)	1071 (34.5)	1119 (36.1)	1071 (34.5)	*P* = 0.001
csDMARDs use reported by physician					
Visits (*n*)	3101	3101	3101	3101	3101
Yes (%)	2486 (80.2)	2439 (78.7)	2486 (80.2)	2439 (78.7)	*P* = 0.003
Janus kinase inhibitor use reported by physician					
Visits (*n*)	3101	3101	3101	3101	3101
Yes (%)	336 (10.8)	425 (13.7)	336 (10.8)	425 (13.7)	<0.0001
CS use reported by physician					
Visits (*n*)	3101	3101	3101	3101	
Yes (%)	715 (23.1)	700 (22.6)	715 (23.1)	700 (22.6)	*P* = 0.52

bDMARDs: biologic DMARDs; COVID-19: coronavirus disease 2019; csDMARDs: conventional synthetic DMARDs.

Table 2 highlights the PROs before and during the COVID-19 pandemic. Supplementary Fig. S3, available at *Rheumatology Advances in Practice* online, outlines the changes graphically. There was a statistically significant improvement in fatigue and in self-reported DASs (RADAI) during the pandemic (Table 2). Patients also reported a significant decrease in csDMARD and CS usage and a significant increase in JAK inhibitor usage.

**Table 2.** Patient reported outcomes one year before and during first year of coronavirus disease 2019 (15 March 2020)

*n* = 709	Within 1 year before COVID-19	Within 1 year after COVID-19	Paired comparison		
			Before	After	Difference, *P*-value
Number of interview visits per patient					
*n*	709	709	709	709	709
Mean (s.d.)	2.01 (52.0)	1.83 (0.46)	2.01 (52.0)	1.83 (0.46)	−0.18 (0.66), <0.0001
Total visits per patient					
*n*	709	709	–	–	–
One visit, *n* (%)	65 (9.2)	145 (20.5)	–	–	–
Two visits, *n* (%)	574 (81.0)	542 (76.5)	–	–	–
Three or more visits, *n* (%)	70 (9.8)	22 (3.0)	–	–	–
Time between visits for patients with more than one visit, days					
*n*	644	564	515	515	515
Mean (s.d.)	173.6 (31.4)	182.6 (18.7)	171.8 (32.4)	184.8 (15.8)	13.0 (36.4), <0.0001
HAQ-DI (0–3)					
*n*	709	709	706	706	706
Mean (s.d.)	0.91 (0.75)	0.90 (0.74)	0.91 (0.75)	0.90 (0.74)	−0.01 (0.59), 0.70
HAQ pain (0–3)					
*n*	709	709	705	705	705
Mean (s.d.)	0.93 (0.76)	0.89 (0.78)	0.93 (0.76)	0.89 (0.78)	−0.04 (0.59), 0.05
Fatigue (0–10)					
*n*	709	709	706	706	704
Mean (s.d.)	3.81 (2.87)	3.32 (2.89)	3.80 (2.87)	3.32 (2.89)	−0.48 (2.42), <0.0001
Sleep (0–10)					
*n*	709	709	706	706	706
Mean (s.d.)	2.92 (2.95)	2.77 (2.95)	2.91 (2.95)	2.77 (2.95)	−0.14 (2.56), 0.14
Depression/anxiety (0–3)					
*n*	709	709	706	706	706
Mean (s.d.)	1.16 (0.35)	1.18 (0.37)	1.15 (0.35)	1.18 (0.37)	0.02 (0.36), 0.07
RADAI morning stiffness duration (0–10)					
*n*	709	709	706	706	706
Mean (s.d.)	2.34 (2.66)	2.12 (2.51)	2.34 (2.66)	2.11 (2.52)	−0.23 (2.53), 0.02
RADAI (0–10)					
*n*	709	709	706	706	706
Mean (s.d.)	2.47 (1.95)	2.30 (1.89)	2.46 (1.95)	2.29 (1.89)	−0.17 (1.40), 0.002
EQ5D EuroQoL (0–1)					
*n*	709	709	706	706	706
Mean (s.d.)	0.79 (0.17)	0.80 (0.19)	0.79 (0.17)	0.80 (0.19)	0.01 (0.14), 0.22
Medication number reported by patient					
*n*	672	660	653	653	653
Mean (s.d.)	1.40 (0.61)	1.36 (0.63)	1.44 (0.59)	1.37 (0.63)	−0.07 (0.50), <0.0001
bDMARDs use reported by patient					
Visits (*n*)	1495	1495	1495	1495	1495
Yes (%)	440 (29.4)	425 (28.4)	440 (29.4)	425 (28.4)	*P* = 0.16
csDMARDs use reported by patient					
Visits (*n*)	1495	1495	1495	1495	1495
Yes (%)	1152 (77.1)	1079 (72.2)	1152 (77.1)	1079 (72.2)	*P* < 0.0001
Janus kinase inhibitor use reported by patient					
Visits (*n*)	1495	1495	1495	1495	1495
Yes (%)	160 (10.7)	191 (12.8)	160 (10.7)	191 (12.8)	*P* = 0.0002
CS use reported by patient					
Visits (*n*)	1495	1495	1495	1495	1495
Yes (%)	231 (15.5)	202 (13.5)	231 (15.5)	202 (13.5)	*P* = 0.0002

bDMARDs: biologic DMARDs; COVID-19: coronavirus disease 2019; csDMARDs: conventional synthetic DMARDs; EQ5D EuroQoL: European quality of life 5-dimension questionnaire HAQ-DI: health assessment questionnaire disability index; RADAI: RA disease activity index.

A sub-analysis was performed, limiting the comparison to in-person assessments within the two study periods (Supplementary Table S1, available at *Rheumatology Advances in Practice* online). There were increases in some of the disease activity measures (patient global assessment, SDAI, DAS-28 and ESR). bDMARD use was decreased, and both JAK inhibitor and CS use were increased significantly during the COVID-19 pandemic.

## Discussion

In this study of >1500 RA patients, we found no significant negative impact on disease activity or PROs during the COVID-19 pandemic. To the best of our knowledge, this is the first study to examine the effects of the COVID-19 pandemic on patients with RA on disease activity, medication changes and PROs. Our study and analysis were initiated 1 year after the COVID-19 pandemic started. Therefore, the objective and subjective measures were limited to the first year. Although this might be seen as a limitation, the first year of the pandemic had the main periods of lockdown and, as such, was the time period where patient care was significantly modified.

Differences were seen in the average number of visits and time between visits. However, the differences themselves were not clinically significant. This was true for the average changes in swollen joint counts and tender joint counts. Overall, the DASs in both study periods remained low (CDAI, SDAI and DAS-28), or patients performed slightly better (RADAI). These findings were similar to the results of a study conducted by Glintborg *et al.* [8], who evaluated the impact of the COVID-19 pandemic on treat-to-target strategies and physical consultations in >7000 patients with inflammatory arthritis. They found that overall, PROs and remission rates remained stable despite the reduction in consultations in person. Furthermore, a study examining the effects of COVID-19 on care for those with JIA found no delays or significant differences in disease activity, disability or quality of life scores before and during the pandemic [9].

The PRO scores for mental, social and physical health were either stable or improved during the COVID-19 pandemic, with non-statistically significant improvements in HAQ-DI, HAQ-pain and sleep scores. Improvements in PROs might be explained by changes in patients’ lifestyles, such as being able to work from home. Other studies have found that the time of the pandemic resulted in different outcome measures. Gavigan *et al.* [10] found that the scores for mental health fluctuated widely depending on the month of the pandemic. They found that physical health assessment scores remained unchanged and that overall mental, social and physical health scores improved during the summer of 2021, when there was the widespread availability of vaccines. It is possible that patients experienced greater amounts of mental stress during periods with significant impacts on the health-care system, such as during lockdowns or periods of increasing incidence rates. However, the only mental health aspect that is included in our registry is the presence of depression, and we did not find any statistically significant changes in depression before and during the first year of the COVID-19 pandemic.

Some medication changes were statistically significant during the COVID-19 pandemic. Decreases in csDMARD use were accompanied by increases in JAK inhibitor use, and bDMARD usage before and during the pandemic remained high, although there was a noticeable decrease. Decreases in csDMARD and bDMARD use with increases in JAK inhibitor use might be explained by rheumatologists attempting to achieve disease control with an oral-based therapy instead of treatment modalities that require infusions at a clinic-based site when csDMARDs fail. Patient access to infusion centres was affected early during the pandemic. Furthermore, JAK inhibitors have been shown to be an effective treatment modality for severe COVID-19 infections [11]. Therefore, physicians might have perceived this class as safer for RA patients with active disease. These data represent the first half of the pandemic. Since then, there has been an important publication, the ORAL-Surveillance, a study showing the higher risk of cardiovascular diseases with JAK inhibitors in high-risk patients compared with biologics [12]. This information might have impacted the physicians’ therapy decisions in the second half of the pandemic.

It is essential to recognize that our work has several limitations. The study population focuses on patients with RA residing within Ontario, for which a specific strategy was undertaken with regard to lockdown and re-opening. This might not be representative of the approach that other countries globally took for the COVID-19 pandemic. The Premier of Ontario declared the first lockdown on 23 March 2020, forcing the closure of all non-essential businesses across the province for 14 days. This was followed by an extended period of using state-of-emergency measures until 15 July to facilitate a gradual reopening of the province. The reopening of regions across Ontario did not occur at the same time and was determined by the number of active/emerging cases in the area. Toronto, the largest city in Ontario, had one of the longest lockdown periods, with restrictions to services lasting >360 days. However, not all regions in Ontario had these constraints in place, and some areas had them removed sooner by comparison. Given the heterogeneity of each region having different reopening time lines, we did not explore the impact of this on the results. There is a risk of selection bias, because patients enrolled in OBRI who continued to attend in-person or interview assessments during the COVID-19 pandemic do not necessarily represent all patients with RA. These patients might be more willing to participate in their care and are more invested in their disease management. As a result, non-adherence to medical therapy increases disease activity, and PROs might be underestimated. Furthermore, given that this was an observational study, we were limited by missing data, particularly on disease activity measures owing to some patients being unable to attend appointments because of the pandemic. Moreover, our dataset did not include information on work status. During the COVID-19 pandemic in Ontario, the majority of individuals, except for essential workers, were working from home; therefore, we might not be able to evaluate the impact of work status on COVID-19 infections and disease outcomes.

The strengths of our study include having real-world patients, which enhances generalizability to cohorts with similar patient characteristics. We also examined several different disease activity indices and PROs and found similar results.

In conclusion, patients with RA during the first year of the COVID-19 pandemic either had no clinically significant change in their disease activity and PROs or performed slightly better in Ontario, Canada. Whether the impacts of the pandemic are increasing over time requires longer-term follow-up.

## Supplementary Material

rkad042_Supplementary_Data

## Data Availability

The data underlying this article will be shared on reasonable request to the corresponding author.
