# Supplementary material for: Impact of the COVID-19 pandemic on patients with rheumatoid arthritis: data from the Ontario Best Practices Research Initiative (OBRI)
Source: Rheumatol Adv Pract. 2023 Apr 26;7(2):rkad042. doi: 10.1093/rap/rkad042 (PMC10172034; doi:10.1093/rap/rkad042)
Supplement: rkad042_Supplementary_Data [file rkad042_supplementary_data.docx]

**Supplementary Figure S1. Cohort flowchart**

**
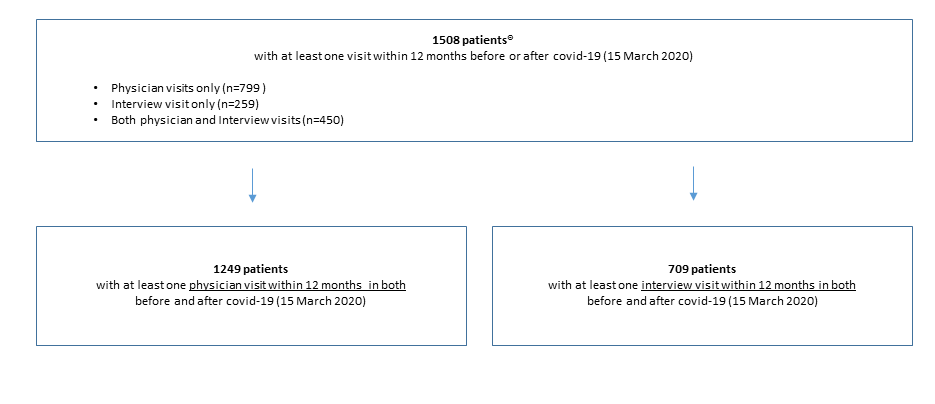
**

**Supplementary Figure S2. Patient reported outcomes one year before and during first year of Covid-19 (15 March 2020)**

**Supplementary Figure S3. Physician disease activity measures one year before and during first year of Covid-19 (15 March 2020)**

| **Supplementary Table S1. Disease activity measures by physician before and after covid-19 limited to patients with in-person visits (15 March 2020)** | | | | | |
| --- | --- | --- | --- | --- | --- |
| Patients (N=415) | Within one year before COVID-19 | Within one year after COVID-19 | Paired comparison | | |
|  |  |  | Before | After | Difference,  p-value |
| Time (days) between in-person visits for patients with more than one visit |  |  |  |  |  |
| N | 230 | 230 | 230 | 230 | 230 |
| Mean ± SD | 66.9± 34.8 | 107.7± 54.9 | 66.9± 34.8 | 107.7± 54.9 | 40.8 (64.6), <0.0001 |
| Swollen Joint Counts (0-10) |  | | |  |  |
| N | 411 | 374 | 371 | 371 | 371 |
| Mean ± SD | 1.92 ± 2.99 | 1.85± 3.19 | 1.88 ± 2.95 | 1.82 ± 3.18 | -0.06 (3.31), 0.74 |
| Tender Joint Counts (0-10) |  | | |  |  |
| N | 411 | 371 | 368 | 368 | 368 |
| Mean ± SD | 2.22 ± 3.26 | 2.22 ± 3.70 | 2.03 ± 2.91 | 2.20 ± 3.70 | 0.17 (3.87), 0.41 |
| Patient Global assessment (0-10) |  | | |  |  |
| N | 363 | 253 | 243 | 243 | 243 |
| Mean ± SD | 3.13 ± 2.45 | 3.59 ± 2.59 | 3.06 ± 2.45 | 3.55 ± 2.61 | 0.49 (2.45), 0.002 |
| Physician Global assessment (0-10) |  | | |  |  |
| N | 352 | 259 | 251 | 251 | 251 |
| Mean ± SD | 1.76 ± 1.94 | 1.88 ± 2.10 | 1.62 ± 1.85 | 1.84 ± 2.07 | 0.22 (2.05), 0.09 |
| Clinical Disease Activity Index (0-76) |  | | |  |  |
| N | 369 | 260 | 252 | 252 | 252 |
| Mean ± SD | 9.24 ± 8.76 | 9.65 ± 9.18 | 8.77 ± 8.05 | 9.54 ± 9.19 | 0.77 (9.32), 0.19 |
| Simplified Disease Activity Index (0.1-86) |  | | |  |  |
| N | 327 | 224 | 207 | 207 | 207 |
| Mean ± SD | 9.91 ± 8.95 | 10.7 ± 9.98 | 9.07 ± 8.29 | 10.72 ± 10.2 | 1.65 (9.76), 0.02 |
| Disease Activity Score-28 (0-9.4) |  | | |  |  |
| N | 344 | 249 | 230 | 230 | 230 |
| Mean ± SD | 3.04± 1.30 | 3.22 ± 1.30 | 2.92 ± 1.26 | 3.24 ± 1.31 | 0.32 (1.23), <0.0001 |
| Erythrocyte Sedimentation Rate |  | | |  |  |
| N | 330 | 285 | 266 | 266 | 266 |
| Mean ± SD | 19.9 ± 17.1 | 22.0 ± 18.8 | 20.2 ± 17.5 | 22.2 ± 19.1 | 2.00 (13.3), 0.01 |
| C-Reactive Protein |  | | |  |  |
| N | 360 | 316 | 292 | 292 | 292 |
| Mean ± SD | 7.03 ± 11.2 | 7.92 ± 12.3 | 6.76 ± 10.9 | 7.66 ± 16.1 | 0.90 (14.6), 0.29 |
| Medication number reported by physician |  |  |  |  |  |
| N | 408 | 410 | 407 | 407 | 407 |
| Mean ± SD | 1.52 ± 0.63 | 1.60 ± 0.67 | 1.52 ± 0.63 | 1.60 ± 0.68 | 0.08 (0.53), 0.005 |
| bDMARDs use reported by physician |  |  |  |  |  |
| Visits (N) | 1007 | 1007 | 1007 | 1007 | 1007 |
| Yes (%) | 343 (34.1) | 317 (31.5) | 343 (34.1) | 317 (31.5) | P=0.004 |
| csDMARDs use reported by physician |  |  |  |  |  |
| Visits (N) | 1007 | 1007 | 1007 | 1007 | 1007 |
| Yes (%) | 780 (77.5) | 775 (77.0) | 780 (77.5) | 775 (77.0) | P=0.80 |
| JAK inhibitor use reported by physician |  |  |  |  |  |
| Visits (N) | 1007 | 1007 | 1007 | 1007 | 1007 |
| Yes (%) | 132 (13.1) | 190 (18.9) | 132 (13.1) | 190 (18.9) | P=<0.0001 |
| Steroid use reported by physician |  |  |  |  |  |
| Visits (N) | 1007 | 1007 | 1007 | 1007 | 1007 |
| Yes (%) | 280 (27.8) | 350 (34.8) | 280 (27.8) | 350 (34.8) | P=<0.0001 |
